# The Efficacy of Music Intervention in Patients with Cancer Receiving Radiation Therapy: A Systematic Review and Meta-Analysis

**DOI:** 10.3390/cancers17040691

**Published:** 2025-02-18

**Authors:** Hsiao-Hsuan Chen, Chia-Hsuan Lai, Yu-Jen Hou, Liang-Tseng Kuo

**Affiliations:** 1 Department of Radiation Therapy, Chang Gung Memorial Hospital, Chiayi 613, Taiwan; hsuan6801@gmail.com (H.-H.C.); chiahsuan7092@gmail.com (C.-H.L.); 2 Department of Physical Medicine and Rehabilitation, Chang Gung Memorial Hospital, Chiayi 613, Taiwan; pinging791207@yahoo.com.tw; 3 Department of Sports Medicine, Landseed International Hospital, Taoyuan 320, Taiwan

**Keywords:** anxiety, depression, fatigue, music intervention, radiation therapy, cancer

## Abstract

**Background:** Music intervention (MI) is a promising complementary therapy for alleviating psychological distress in patients with cancer undergoing radiotherapy. This systematic review and meta-analysis assessed the efficacy of MI in reducing anxiety, depression, and fatigue in these patients. **Methods:** A comprehensive search was conducted in MEDLINE, EMBASE, the Cochrane Central Register of Controlled Trials (CENTRAL), ClinicalTrials.gov, and the International Clinical Trials Registry Platform from inception to 9 January 2025. We included randomized controlled trials (RCTs) and cohort studies investigating MI’s impact on psychological outcomes in patients with cancer receiving radiotherapy in this review. The study quality was appraised using the Cochrane Risk of Bias 2.0 for RCTs and the ROBINS-I tool for cohort studies. A meta-analysis was performed using a random-effects model in Review Manager 5.4. **Results:** A total of 13 studies (11 RCTs and 2 cohort studies) with 1073 participants were included. The pooled analysis revealed a significant reduction in anxiety (mean difference [MD]: −3.53, 95% confidence interval [CI]: −5.98 to −1.07, *p* = 0.003), a non-significant effect on depression (MD: −1.12, 95% CI: −3.25 to 1.01, *p* = 0.29), and a significant improvement in fatigue (MD: −15.88, 95% CI: −28.19 to −3.57, *p* = 0.01). A subgroup analysis based on intervention type indicated that music medicine (MM) was more effective in reducing anxiety compared to music therapy (MT). **Conclusions:** The findings of this study suggest that MI, particularly MM, may be beneficial in alleviating anxiety and fatigue in patients with cancer undergoing radiotherapy, but its impact on depression remains inconclusive. Future studies should employ standardized methodologies and rigorous RCT designs to validate these findings.

## Simple Summary

Music intervention (MI) has been recognized as an effective method for alleviating mental health issues in patients. However, the efficacy of MI in patients receiving radiation therapy remains unclear. Significant improvement in the State-Trait Anxiety Inventory score was noted in the music group (mean difference [MD]: −3.53; 95% CI −5.98–−1.07). Additionally, significant improvement was observed in the Symptom Distress Thermometer score (MD −1.34; 95% CI −2.22 to −0.47). The impact on depression was inconclusive, although a significant reduction in fatigue was observed. Our results demonstrate that comprehensive MI may be associated with a patient’s psychological improvement in response to radiotherapy.

## 1. Introduction

According to the latest data released by the World Health Organization (WHO) in February 2024, an estimated 20 million new cancer cases and approximately 9.7 million cancer-related deaths occurred globally in 2022 [1]. Approximately 50% of patients diagnosed with cancer require radiotherapy as a critical component of their treatment regimen [2,3].

Radiotherapy, while essential for cancer treatment, is frequently accompanied by substantial psychological challenges. Patients experiencing repeated treatment sessions confront a complex spectrum of psychological responses, characterized by significant emotional disturbances and heightened vulnerability to psychological stress [4,5]. Empirical research has consistently demonstrated that patients with cancer exhibit an elevated prevalence of non-specific psychological distress and increased susceptibility to comorbid psychiatric disorders [6,7].

Music intervention (MI) has emerged as a promising complementary approach to address these psychological challenges. MI encompasses two distinct modalities: music therapy (MT), involving specialized interventions by trained therapists, and music medicine (MM), which utilizes music-based approaches without formal therapeutic interaction [8]. Despite their similarities, MM and MT differ in several critical aspects that may contribute to their distinct effects in clinical settings. MM is typically administered in a passive listening format, allowing patients to engage with music without requiring active participation. In contrast, MT is delivered by a certified music therapist and involves structured activities such as guided improvisation, songwriting, and lyric analysis.

Methodological differences between these two approaches may also account for their varying degrees of efficacy. MM studies often employ standardized intervention protocols with controlled music selection and exposure duration, leading to more consistent results. On the other hand, MT interventions vary depending on patient needs and therapist expertise, introducing potential variability in outcomes. Additionally, the immediate physiological relaxation effect observed in MM—such as reduced cortisol levels—may explain its stronger impact on anxiety reduction, while MT’s benefits may emerge gradually over multiple sessions.

These distinctions underscore the importance of further research to clarify the relative efficacy of MM and MT in different psychological domains, particularly within the context of radiotherapy. Multiple studies have highlighted the potential therapeutic benefits of MI, including reductions in anxiety and stress levels, improvements in mood states, the enhancement of cognitive functioning, the promotion of neural plasticity, and the mitigation of treatment-related psychological distress [9,10,11,12].

The neurobiological mechanisms underlying MI suggest multifaceted physiological impacts, including the attenuation of cortisol secretion, the stimulation of dopamine release, the modulation of emotional states, and the refinement of interpersonal neural networks [13,14,15,16].

Theoretical frameworks suggest that MI’s efficacy lies in its potential to modulate emotional states [17,18,19,20,21,22,23,24]. Music has been widely integrated into diverse therapeutic approaches, including radiation therapy, with the aim of mitigating anxiety levels. Despite these promising indications, the existing literature presents a research gap. Current evidence regarding the efficacy of MI for patients with cancer undergoing radiotherapy remains inconclusive and fragmented. The primary aim of this study is to assess the effectiveness of MI in mitigating emotional disturbances, particularly anxiety, depression, and fatigue, which are commonly experienced by individuals receiving radiotherapy.

## 2. Materials and Methods

### 2.1. Eligibility Criteria

This systematic review included studies investing the efficacy of music intervention on adult patients with cancer undergoing radiation therapy. The study designs included RCTs, controlled clinical trials, comparative studies, and cohort studies. Our search methodology adhered to the PICO framework as follows: the population (P) constituted patients with cancer within radiation oncology settings (we only included patients aged 18 years or older in our discussion); the intervention (I) comprised music interventions; the comparison (C) involved non-music interventions; and the outcomes (O) centered on psychological improvements such as anxiety, depression, and fatigue.

### 2.2. Literature Search Strategy

MEDLINE, EMBASE, and the Cochrane Central Register of Controlled Trials (CENTRAL) were searched for relevant studies from inception to 9 January 2025. Manual searches were performed for relevant journal articles and conference abstracts in the International Clinical Trials Registry Platform and ClinicalTrials.gov. All available languages were included in the review. The search methodology, encompassing manual searches of reference lists and conference materials, is detailed in Figure S1. Subject matter experts were contacted for additional information where necessary data were unavailable.

### 2.3. Protocol and Registration

This systematic review was conducted and reported in accordance with the PRISMA 2020 guidelines [25] (Table S1) and was registered in the PROSPERO database (CRD42024533279). 

### 2.4. Study Selection

Two reviewers (H-H, C, and C-H, L) reviewed the full-text versions of the remaining articles. In cases of disagreement, a third reviewer (L-T, K) was consulted.

### 2.5. Data Extraction

We utilized a prespecified datasheet to extract data from the included studies. We focused on assessing anxiety, depression, and fatigue statuses before and after the music intervention. Studies reporting outcomes related to pain were excluded from this analysis. This data extraction process was independently performed by two authors (H-H, C and C-H, L), with the third author (Y-J, H) verifying accuracy. When necessary data were absent, we contacted the authors for additional information, including specific study details.

### 2.6. Assessment of Risk of Bias

The Cochrane Risk of Bias 2.0 (RoB 2.0) tool [26] and Risk of Bias In Nonrandomized Studies of Interventions tool (ROBINS-I) [27] were individually used to assess the risk of bias in RCTs and nonrandomized studies. Two reviewers (H-H, C and Y-J, H) assessed the risk of bias for each included study. Disagreements were resolved by discussion and, if necessary, consultation with a third reviewer (L-T, K). The quality of the included studies was evaluated via the Cochrane Risk of Bias 2.0 by two independent investigators (H-H, C and Y-J, H), who assessed the studies as having “low risk”, “some concerns”, or “high risk” on the basis of potential bias in the following domains: randomization process, intended intervention, missing outcome data, measurement of the outcome, and selection of reported result. Any disagreements were resolved through discussion and consensus with a third investigator (L-T, K).

### 2.7. Statistical Analysis

For the meta-analysis, Review Manager version 5.4 from the Nordic Cochrane Collaboration was utilized. We chose a random-effects approach to synthesize the data because of the inherent heterogeneity of the studies. Continuous outcomes were reported using mean differences (MDs) with 95% confidence intervals (CIs). The I^2^ statistic was used to assess the statistical heterogeneity across studies, considering I^2^ values over 50% as substantial heterogeneity. Additionally, a subgroup analysis was conducted to discern differences between music therapy and music medicine interventions. Publication bias was assessed by a funnel plot if the number of included studies was more than 10 [28].

## 3. Results

### 3.1. Search and Selection Process

**Figure 1:**
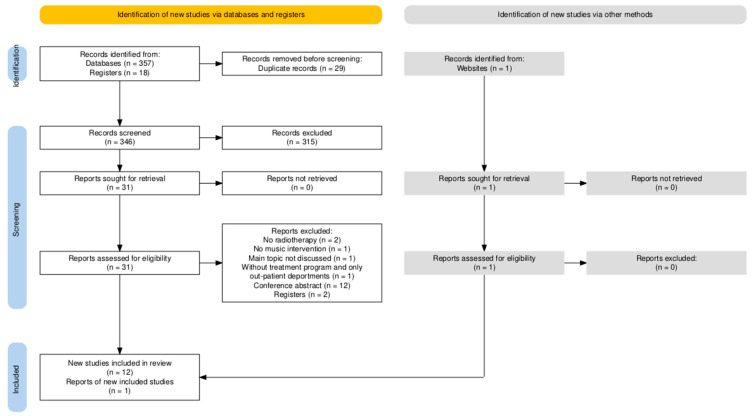
A PRISMA flowchart of the research process for the selection of the included studies.

The PRISMA flow diagram (Figure 1) shows our search process. A total of 375 articles were identified from the databases and trial registration websites. An additional Taiwanese study was added from local data. After 29 duplicates were removed, 346 articles were subjected to title and abstract reviews. Ultimately, 11 randomized controlled trials and two cohort studies, with a total of 1073 participants, were selected for inclusion. The included articles utilized various assessment scales, including the State-Trait Anxiety Inventory (STAI), Symptom Distress Thermometer (SDT), Hospital Anxiety and Depression Scale (HADS), Hamilton Anxiety Rating Scale (HAM-A), Self-Rating Anxiety Scale (SAS), Beck Anxiety Inventory (BAI), and Brief Symptom Rating Scale-5 (BSRS-5) to evaluate anxiety; the Functional Assessment of Cancer Therapy-Fatigue (FACT-F) to assess fatigue; and the Beck Depression Inventory (BDI) to evaluate depression.

### 3.2. The Characteristics and Clinical Parameters of the Included Studies

Table 1 and Table 2 present the characteristics and clinical parameters of the included studies. Table 1 focuses on the characteristics of these music interventions, categorizing them by authors and publication years. It comprehensively details each intervention, including the style of music used, the method of delivery, whether it is classified as music medicine or therapy, and the schedule of the intervention. Additionally, this table considers patient preferences for music. Table 2 provides an extensive overview of the studies featured in this systematic review, with a focus on the application of music interventions in radiation therapy for patients with cancer. It itemizes these studies by author, country of origin, and study design, providing specifics on the diagnosis, setting, and demographics of the participants. This table also shows the type of music intervention used in each study and the mean age of the participants. Furthermore, it enumerates the outcomes measured in these studies, which include factors such as anxiety, depression, fatigue, and overall quality of life.

### 3.3. Risk of Bias Assessment

Among the 11 trials used for analysis in this RCT [12,13,29,30,31,32,33,34,35,36,38], all were determined to have a high risk of bias (Table S2) because the outcomes were measured using self-report questionnaires completed by the subjects. Participants in the music intervention group may have experienced greater relaxation due to their awareness of receiving music therapy, which could have psychologically influenced their self-reported outcomes. Additionally, the process of allocation concealment was not described, and there was an uneven number of stage 0 participants at baseline between the two groups. The fully reported results are likely to have been selected based on the outcomes.

Among the two cohort studies [10,38] used for analysis (Table S3), the results were deemed critical because the authors did not account for potential confounding factors that could influence the outcomes. Additionally, the measurement and reporting biases were considered serious, as the outcomes were assessed through self-report questionnaires completed by the subjects. The participants in the music intervention group might have felt more relaxed, and this psychological effect could have influenced their self-reported results. The fully reported results are likely to have been selected based on the results.

### 3.4. Main Outcomes

#### 3.4.1. The Impact of Music Intervention on Anxiety Levels

**Figure 2:**
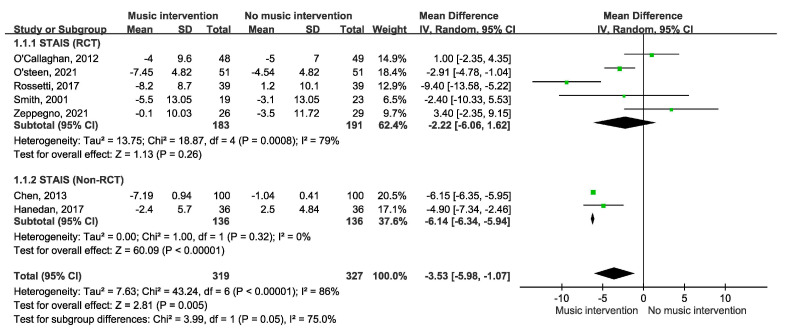
Forest plot of anxiety outcomes using STAIS in five RCTs and two cohort studies [12,13,29,32,35].

**Figure 3:**
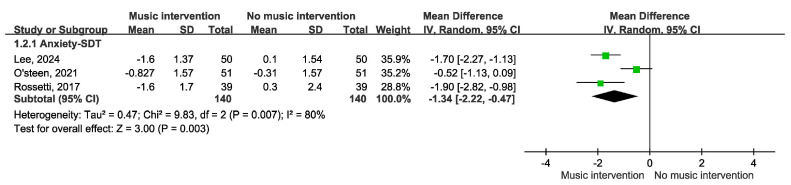
Forest plot of anxiety outcomes using SDT in three RCT studies [12,13,38].

**Figure 4:**
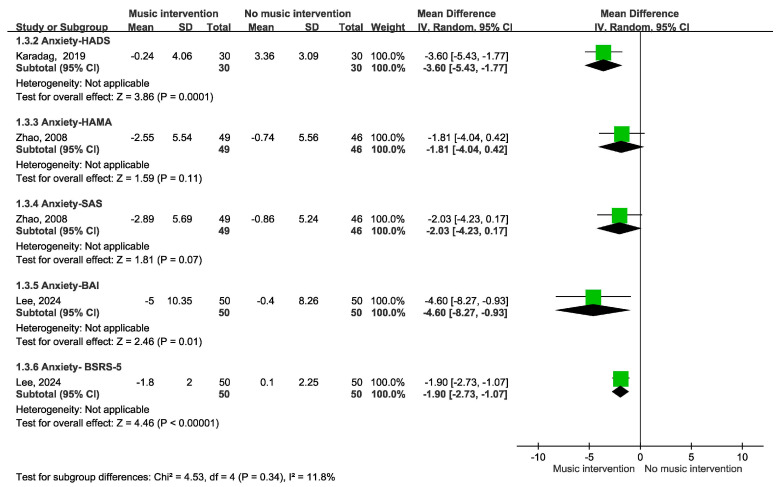
Forest plot of anxiety—additional rating scale analysis [31,34,38].

**Figure 5:**
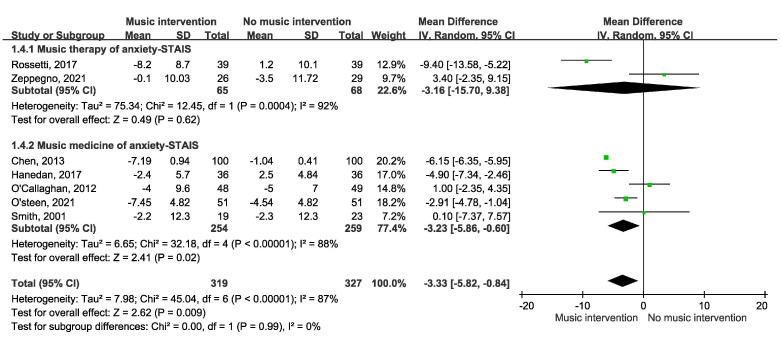
Forest plot comparing anxiety outcomes using STAIS between music therapy (MT) and music medicine (MM) [11,12,13,29,30,32,35].

**Figure 6:**
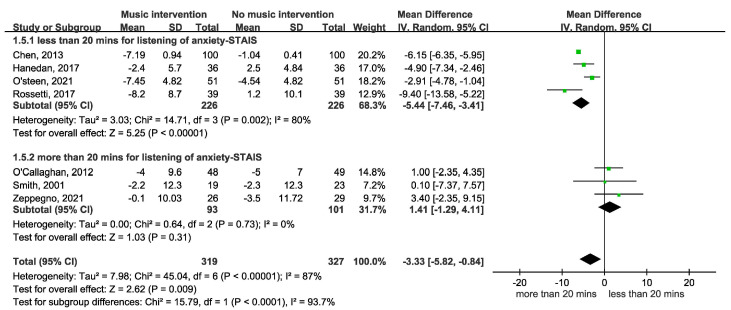
A forest plot showing that listening to music for less than 20 min significantly reduced anxiety levels, as assessed using the STAI scale [11,12,13,29,30,32,35].

Figure 2 depicts the impact of music intervention on anxiety levels among patients with cancer undergoing radiation therapy, as assessed via the STAI-S scale [39]. Notably, a statistically significant decrease in anxiety was evident, with a mean difference of −3.53 (95% CI −5.98 to −1.07, *p* = 0.003). Five randomized controlled trials (RCTs) were included. However, the effect of music intervention on anxiety levels during RT was not statistically significant, with a mean difference of −2.22 (95% CI −6.06 to 1.62), accompanied by substantial heterogeneity (I^2^ = 79%). Conversely, the findings from two cohort studies reveal a favorable impact of music intervention, yielding a mean difference of 6.14 (95% CI −6.34 to −5.94) without heterogeneity (I^2^ = 0%). Our detailed meta-analysis of seven studies, including five randomized controlled trials (RCTs) and two non-RCTs employing the STAI-S scale, revealed that among the RCTs involving 374 participants, there were no significant differences in anxiety levels immediately after RT. However, improvements were observed over longer durations of two weeks to three months in the studies by Zeppagno and Raglio [35,36]. These findings suggest a positive effect of music interventions on patient anxiety, highlighting the need for larger-scale studies to provide more definitive evidence. As shown in Figure 3, a significant improvement was observed in the Symptom Distress Thermometer (SDT) score (MD −1.34; 95% CI −2.22 to −0.47, *p* = 0.003). Furthermore, anxiety levels were assessed via additional anxiety scales, including the HADS, HAMA, SAS, BAI, and BSRS-5. However, owing to the variability inherent in the scales employed, an amalgamated analysis of these outcomes is not presented in Figure 4. A subgroup analysis revealed distinctions between music therapy and music medicine in Figure 5, using the STAI scale to assess anxiety. Music medicine appears to be effective in reducing anxiety, with a mean difference of −3.33 (95% CI −5.82 to −0.84, *p* = 0009). In contrast, the efficacy of music therapy remains inconclusive because of the limited number of participants. An analysis of the data presented in Figure 6 shows that listening to music for less than 20 min significantly reduces anxiety levels, as assessed using the STAI scale, with a notable mean difference of −5.44 (95% CI: −7.46 to −3.41, *p* < 0.00001). In contrast, listening to music for more than 20 min does not significantly impact anxiety, as indicated by a mean difference of 1.14 (95% CI: −1.29 to 4.11, *p* = 0.31).

#### 3.4.2. The Impact of Music Intervention on Depression Outcomes

**Figure 7:**
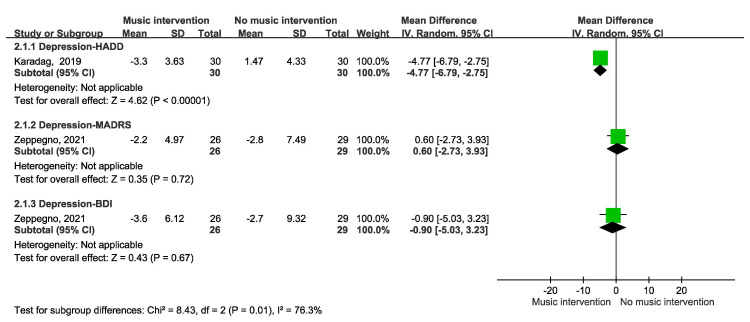
Forest plot depicting depression outcomes [34,35].

Regarding depression outcomes, the relationship between music intervention and depression during radiotherapy remained unclear in two RCTs, as depicted in Figure 7, due to variability in the assessment scales used, including the HADD, MADRS, and BDI.

#### 3.4.3. The Impact of Music Intervention on Fatigue Outcomes

**Figure 8:**
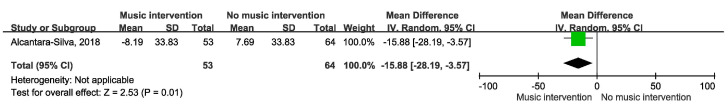
Forest plot of fatigue outcomes showing improvement in FACT-F scale scores [33].

Figure 8, which shows the forest plot for fatigue outcomes, indicates a significant improvement in the FACT-F scale scores, with a mean difference of −15.88 (95% CI, −28.19–−3.57, *p* = 0.01).

## 4. Discussion

In this study, we clearly demonstrate that a music intervention may contribute to patients’ psychological improvement in response to radiotherapy whenever the improvement in depression in patients is not evident. Our analysis included a total of five randomized controlled trials and two nonrandomized controlled trials. All of the trials included 646 patients whose data were published between 2001 and 2024.

Music intervention can be categorized into two primary approaches: music medicine and music therapy. A subgroup analysis revealed distinctions between music therapy and music medicine, as shown in Figure 5. Music medicine appears to be effective in reducing anxiety, while the efficacy of music therapy remains inconclusive due to a limited number of participants. The observed differences in anxiety reduction between MM and MT may be attributed to several critical factors. First, methodological variations play a significant role. MM interventions are typically standardized, ensuring uniform music exposure across participants, whereas MT sessions are tailored to individual patient needs and therapist approaches, introducing variability in outcomes. Second, patient engagement levels differ between the two interventions. MM involves passive listening, which can induce immediate autonomic relaxation. In contrast, MT requires active participation, potentially necessitating prolonged engagement before its therapeutic benefits become evident. Third, the intervention setting may contribute to the observed discrepancies. MM is often delivered in controlled clinical environments, reducing external influences, whereas MT’s effectiveness can be influenced by session dynamics, patient-therapist interactions, and therapist expertise. Finally, study limitations should be considered. The relatively small number of MT studies included in this meta-analysis may have led to an underestimation of its full therapeutic potential. Further large-scale trials are necessary to determine whether MT provides distinct long-term benefits compared to the more immediate effects observed with MM. 

Despite the increasing acknowledgment of the potential benefits of music therapy, its integration into clinical practice remains limited. This gap underscores the necessity for further empirical research to assess the efficacy of music therapist-led interventions rigorously. Such research is crucial to substantiate the therapeutic value of music therapy and facilitate its broader adoption in clinical settings. Notably, our study included only two articles on music therapy [13,35], each involving a relatively small number of participants. This limitation further emphasizes the need for more extensive research to comprehensively evaluate the therapeutic potential of music therapy and to support its broader adoption in clinical settings. However, improvements were observed over longer durations of two weeks to three months in studies by Zeppagno and Raglio [35,36].

These findings suggest a positive effect of music interventions on patient anxiety, highlighting the need for larger-scale studies to provide more definitive evidence. Given the variability in study designs and the heterogeneity in reported outcomes, we recommend the future research on music interventions for radiotherapy and cancer patients should address methodological inconsistencies to enhance reliability and clinical applicability. Standardizing intervention protocols, including music selection, session duration, and delivery methods, is essential for ensuring consistency across studies. Additionally, adopting validated psychological assessment tools such as STAI and HADS scores can improve comparability of outcomes. Controlling for confounding factors, such as patient demographics, cancer type, and concurrent treatments, will help minimize bias and strengthen study validity. Expanding research to diverse patient populations, including different ethnicities and age groups, can improve the generalizability of findings. Furthermore, long-term follow-up is necessary to assess sustained psychological benefits, as most existing studies focus only on short-term effects. High-quality RCTs with well-matched control groups remain crucial for establishing MI as an evidence-based adjunctive therapy. Addressing these gaps will help generate robust evidence supporting the integration of MI into standard cancer care. The demanding nature of clinical work underscores the importance of supporting patients’ psychological states during radiotherapy, which can strengthen the rationale and confidence of clinical staff in providing music interventions.

The current evidence indicates a high risk of bias, especially due to the challenges in blinding participants in assessments of psychological states, which often rely on self-reported scales. This concern is echoed in Bradt’s findings [40], highlighting the potential of music interventions to positively affect anxiety, depression, hope, pain, and fatigue in adults with cancer. However, many trials present a high risk of bias, with the quality of evidence ranging from low to very low. Consequently, more rigorous and well-designed studies are necessary to validate these findings and enhance the reliability of the evidence supporting music interventions.

Previous literature [37] indicates that interventions lasting 15–40 min can effectively promote auditory system adjustment and neural plasticity, leading to significant effects from specific auditory stimuli. The recommended duration for music intervention should be at least 15–40 min. However, based on the data we combined from Figure 6, listening to music for less than 20 min significantly reduces anxiety levels, with a mean difference of −5.44. In contrast, listening to music for more than 20 min does not show a significant effect, with a mean difference of 1.41. This analysis suggests that shorter durations of music listening (less than 20 min) may be more effective in reducing anxiety than longer sessions.

The effects of the training are still observable in subsequent tests, indicating that even short-term interventions can have lasting impacts on brain function. These findings can serve as a reference for the dosage and effectiveness of clinical music interventions. One study [35] showed that music therapy did not improve anxiety immediately after the completion of radiation therapy, but an anxiety reduction was observed three months posttreatment. Increasing the dosage and frequency of music intervention might alleviate distress [29], emphasizing the role of high-quality educational content and supportive caregivers in mitigating psychological distress. Patient feedback reveals diverse preferences for music, with some experiencing adverse reactions due to machine noise and uncomfortable positions during treatment. This highlights the importance of empathy and support in achieving therapeutic goals.

Two articles concerning depression were identified, but they employed different assessment tools. One article reported improvements, whereas the other did not, leading to a lack of consensus on the topic.

Previous studies, mostly small-scale, have revealed significant improvements in the psychological state of patients undergoing radiation therapy with music intervention in Asia. This aligns with clinical experiences, suggesting that cultural background may enhance the effectiveness of music interventions. Patients often request music during treatment when not initially provided, indicating a proactive desire for such interventions. Future research should aim for larger-scale studies to provide more objective data on this topic.

Even if the results of the study do not provide strong evidence for the improvement of psychological states, empathizing with the patient’s mood and offering music, which does not incur high costs, remains a viable option.

This meta-analysis has several limitations, including the relatively small number of included studies, resulting in insufficient data for some subgroups. Additionally, the type of cancer and duration of treatment may affect the impact of music interventions on patients. We aim to conduct an updated meta-analysis when more studies become available. The incorporation of multiple depression scales (e.g., the STAI-S, SDT, HADS, HAMA, and SAS) complicates the overall evaluation of data, underscoring the need for future clinical trials to address the issue of an insufficient number of cases included.

## 5. Conclusions

The music intervention group showed an improvement in anxiety, as indicated by the STAI-S and SDT scores, highlighting the potential benefits of music during radiation treatment. Although there was a significant reduction in fatigue levels, the impact of music on depression remains unclear. These results demonstrate the value of music as an adjunct therapy in the context of radiation treatment.

## Figures and Tables

**Table 1:** Characteristics of music interventions.

Study (Author, Year)	Music Intervention Details				Patient’s Perspective
	Music Style	Music Delivery: Music/Equipment (Headphones vs. Loudspeakers)	Type Of Music Intervention: MM Or MT/Person Who Chooses Music	Schedule of Intervention Single or Multiple Sessions/ Before or During Treatment/ Duration/Frequency	Preference for Music
Smith et al., 2001 [29]	Rock and roll, big band, country, Western, classical, easy listening, Spanish or religious music; 4–7 tapes were available in each category	Recorded music (CD)/headphones	MM/patients were allowed to select type from category of their choice	Single session/during treatment/30 min/daily	Yes
Clark et al., 2006 [30]	N/A	Recorded music (cassette tape)/headphones	MT/music therapist	Multiple sessions/during treatment course/90 min/2–4 times per week for 4–5 weeks	No
Zhao et al., 2008 [31]	100 songs included Chinese and Western classical music, religious music, yoga music, slow tempo, and easy listening	Recorded music/headphones	MM/patients select from list	Single session/during treatment/30 min/daily	Yes
O’Callahgan et al., 2012 [32]	Self-selection	Recorded music/broadcast	MM/patients bring their own music	Single session/during treatment/27 min/daily	Yes
Chen et al., 2013 [10]	Songs in Mandarin, Mandarin pop, traditional Taiwanese songs, Western music, classical music	Recorded music/headphones	MM/patients bring their own music	Single session/before treatment/15 min/daily	Yes
Uslu et al., 2017 [11]	Turkish folk music, Turkish classical music, Turkish popular music	Recorded music (MP3)/broadcast	MM/patients select from list	Single session/during treatment/12–15 min/daily	Yes
Rossetti et al., 2017 [13]	N/A	Live music	MT/music therapist	Single session/before simulation/20 min/daily	No
Alcantara-Silva et al., 2018 [33]	Baroque, classical, and romantic periods; slow tempo, tone, and regular pulse	Recorded music (iPod)/broadcast	MT/music therapist	Single session/before treatment/30–40 min/2–4 times per week	No
Karadag, Uğur and Çetinayak, 2019 [34]	19 TrioSonatas, relaxing pieces of Bach, rhythm	Recorded music (MP3)/headphones	MM/investigator	Multiple sessions/before treatment/15–40 min/a few times per day, 3–5 days per week	No
Zeppegno et al., 2021 [35]	Italian pop repertoire	Laptop/wireless and bluetooth speakers	MT/music therapist	Single session/during treatment course/60 min/six weekly sessions during 4–5 weeks	No
O’steen et al., 2021 [12]	Music from web-based application	Prerecorded music/ broadcast	MM/select from web-based application	Single session/during treatment/N/A/daily	Yes
Raglio et al., 2021 [36]	Musical pieces with therapeutic purpose	Prerecorded music/earphones	MM/melomics-health algorithm	Multiple sessions/before treatment/15 min/daily	No
Raglio et al., 2021 [36]	Individualized music	Prerecorded music/musical content was designed	MT/music therapist	Multiple session/before treatment/15 min/daily	No
Lee et al., 2024 [37]	360,000 songs, including relaxing piano and violin music	Recorded music (MP3)/bluetooth speaker	MM/MP3 music file database	Single session/during treatment/10–15 min/daily	No

Abbreviations: CD, compact disk; MM, music medicine; MT, music therapy; N/A: not available.

**Table 2:** Study demographics.

Study	Country	Study Design	Cancer Type	Setting	Participants	Age (Mean)	Outcomes
Smith et al., 2001 [29]	USA	RCT	Prostate, lung, head and neck, colorectum, skin squamous cell carcinoma, stomach, and melanoma	Simulation and during RT	MI: 19 No MI: 23	62.2 63.4	Anxiety (STAI)
Clark et al., 2006 [30]	USA	RCT	Prostate, breast, lung, head and neck, gastrointestinal, and gynecological	During RT course	MI: 35 No MI: 28	58.6 56.7	Anxiety (HAD-A) Depression (HAD-D) Pain (NRS) Emotional distress (NRS) Fatigue (POMS)
Zhao et al., 2008 [31]	China	RCT	Lung, colorectum, esophagus, stomach, liver, and breast	Before and after RT	MI: 49	53.6	Anxiety (SAS, HAMA)
					No MI 46	54.6	
O-Callahgan et al., 2012 [32]	Australia	RCT	Prostate, cervix, endometrium, breast, and lung	During RT	MI: 48 No MI: 49	58.0 57.0	Anxiety (STAI)
Chen et al., 2013 [10]	Taiwan	Non RCT	Head and neck, gynecological, breast, digestive tract, lung, and prostate	Before RT	MI: 100 No MI: 100	55.0 55.6	Anxiety (STAI)
Rossetti et al., 2017 [13]	USA	RCT	Breast and head and neck	Before simulation	MI: 39 No MI: 39	58.5 58.0	Anxiety (STAI, SDT)
Uslu et al., 2017 [11]	Turkish	Non RCT	Gastrointestinal and genitourinary	Before and after RT	MI: 36 No MI: 36	N/A N/A	Anxiety (STAI)
Alcantara-Silva et al., 2018 [33]	Brazil	RCT	Breast and gynecological	Before RT	MI: 53 No MI: 63	51.8 52.9	Quality of life (FACT-G) Depression (BDI) Fatigue (FACT-G)
Karadag, Uğur and Çetinayak, 2019 [34]	Turkey	RCT	Breast	During RT	MI: 30 No MI: 30	62.2 56.6	Anxiety (HAD-A) Depression (HAD-D) Comfort (RTCQ)
Zeppegno et al., 2021 [35]	Italy	RCT	Breast	During RT course	MI: 26 No MI: 29	63.0 66.6	Anxiety (STAI) Depression (MADRS, BDI)
O’steen et al., 2021 [12]	USA	RCT	Breast, CNS, gastrointestinal, head and neck, lung, and lymphoma	During RT	MI: 51 No MI: 51	63.0 62.0	Anxiety (STAI, SDT)
Raglio et al., 2021 [36]	Italy	RCT	Breast	Before RT	MI:20 No MI:19	N/A N/A	Anxiety (STAI, PDI)
Lee et al., 2024 [37]	Taiwan	RCT	Head and neck, breast, lung, colorectum, and gynecological	During RT	MI:50 No MI:50	59.9 60.3	Anxiety (BAI-C) Distress (SDT) Mood (BSRS-5)

Abbreviations: BAI-C, Beck Anxiety Inventory; BDI, Beck Depression Inventory; BSRS, Brief Symptom Rating Scale; CNS, central nervous system; FACT-G, functional assessment of cancer therapy; HAD-A, hospital anxiety, and depression–anxiety; HAD-D, hospital anxiety, and depression–depression; HAMA, Hamilton anxiety scale; MADRS, Montgomery-auberge depression rating scale; MI, music intervention; NRS, numeric rating scale; PDI, psychological distress inventory; POMS, profile of mood states; RCT, randomized controlled trial; RT, radiation therapy; RTCQ, radiation therapy comfort questionnaire; SAS, Self-Rating Anxiety Scale; SDT, symptom distress thermometer; STAI, State-Trait Anxiety Inventory.

## Data Availability

All data and materials are publicly available.

## Supplementary Materials

The following supporting information can be downloaded at: https://www.mdpi.com/article/10.3390/cancers17040691/s1, Figure S1: Search methodology; Table S1: PRISMA_2020_checklist; Table S2: ROB2.0 depicts the bias risks in the included RCT studies; Table S3: ROBINS-I. depicts the bias risks in the included cohort studies. Reference [41] are cited in the Supplementary Materials.

## Abbreviations

The following abbreviations are used in this manuscript:

MI	Music Intervention
MD	Mean Different
MT	Music Therapy
MM	Music Medicine
CIs	Confidence Intervals
STAI	State-Trait Anxiety Inventory
SDT	Symptom Distress Thermometer
HADS	Hospital Anxiety and Depression Scale
HAM-A	Hamilton Anxiety Rating Scale
SAS	Self-Rating Anxiety Scale
BAI	Beck Anxiety Inventory
BSRS-5	Brief Symptom Rating Scale-5
FACT-F	Functional Assessment of Cancer Therapy-Fatigue
BDI	Beck Depression Inventory
