# Supplementary material for: The Efficacy of Music Intervention in Patients with Cancer Receiving Radiation Therapy: A Systematic Review and Meta-Analysis
Source: Cancers (Basel). 2025 Feb 18;17(4):691. doi: 10.3390/cancers17040691 (PMC11852407; doi:10.3390/cancers17040691)
Supplement: Supplementary file 1 [file cancers-17-00691-s001.zip › Table S2. ROB2.0 depicts the bias risks in the included RCT studies.pdf]

Table S2. ROB2.0 depicts the bias risks in the included RCT studies.

Publication	Randomisation process	Deviations from intended interventions	Missing outcome data	Measurement of the outcome	Selection of the reported result	Overall bias
Lee, 2024	Low	Low	Low	High	Low	High
Raglio, 2021	Low	Some concerns	Low	High	High	High
O'steen, 2021	High	Low	Low	High	Low	High
Zeppegno, 2021	Low	Low	Low	High	High	High
Karadag, 2019	High	Low	Low	High	High	High
Alcantara-Silva, 2018	Low	High	High	High	Low	High
Rossetti, 2017	Some concerns	Some concerns	Low	High	Low	High
O' Callahgan, 2012	Some concerns	Some concerns	Low	High	Low	High
Zhao, 2008	Some concerns	Some concerns	Low	High	High	High
Clark, 2006	Some concerns	Some concerns	Low	High	High	High
Smith, 2001	Some concerns	Some concerns	Low	High	Low	High
