# Supplementary material for: The Efficacy of Music Intervention in Patients with Cancer Receiving Radiation Therapy: A Systematic Review and Meta-Analysis
Source: Cancers (Basel). 2025 Feb 18;17(4):691. doi: 10.3390/cancers17040691 (PMC11852407; doi:10.3390/cancers17040691)
Supplement: Supplementary file 1 [file cancers-17-00691-s001.zip › Table S3. ROBINS-I. depicts the bias risks in the included cohort studies.pdf]

Table S3. ROBINS-I. depicts the bias risks in the included cohort studies.

Publication	Confounding	Selection of participants	Classification of interventions	Deviations from intended interventions	Missing data	Measurement of outcome	Selection of the reported result	Overall bias
Chen, 2013	Critical	Low	Low	No information	Low	Serious	Serious	Critical
Hanedan, 2017	Critical	No information	Low	No information	Low	Serious	Serious	Critical
