# Supplementary figures and images for: The Efficacy of Music Intervention in Patients with Cancer Receiving Radiation Therapy: A Systematic Review and Meta-Analysis
Source: Cancers (Basel). 2025 Feb 18;17(4):691. doi: 10.3390/cancers17040691 (PMC11852407; doi:10.3390/cancers17040691)

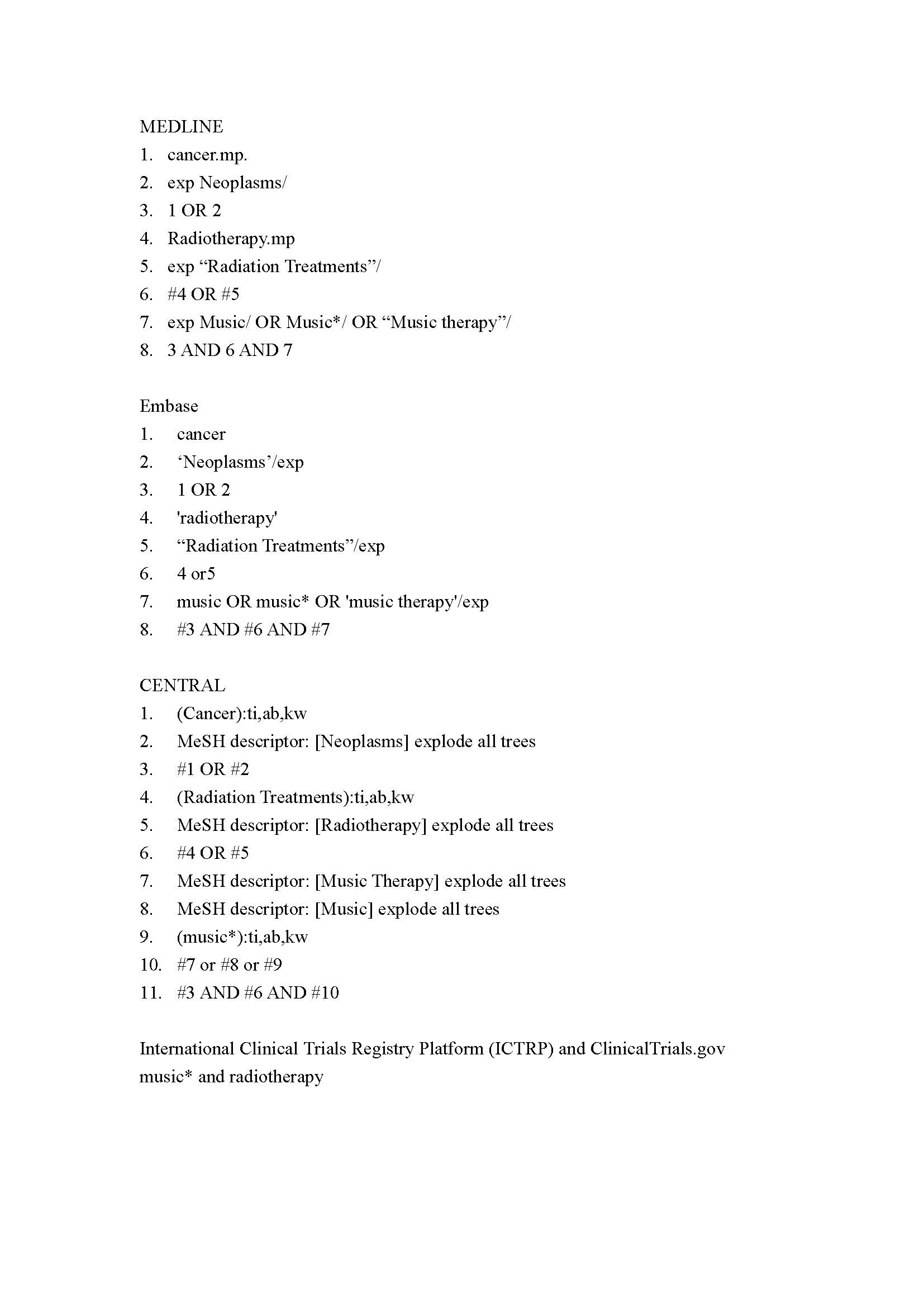

Supplement: Supplementary file 1 [file cancers-17-00691-s001.zip › Figure S1. Search methodology.jpg]
